# Serotonergic Neurons in the Chick Brainstem Express Various Serotonin Receptor Subfamily Genes

**DOI:** 10.3389/fphys.2021.815997

**Published:** 2022-01-17

**Authors:** Toshiyuki Fujita, Naoya Aoki, Chihiro Mori, Eiko Fujita, Toshiya Matsushima, Koichi J. Homma, Shinji Yamaguchi

**Affiliations:** ^1^Department of Biological Sciences, Faculty of Pharmaceutical Sciences, Teikyo University, Tokyo, Japan; ^2^Department of Molecular Biology, Faculty of Pharmaceutical Sciences, Teikyo University, Tokyo, Japan; ^3^Department of Biology, Faculty of Science, Hokkaido University, Hokkaido, Japan

**Keywords:** chick, dorsal raphe nucleus, median raphe nucleus, serotonin receptor, heterogeneity, optic tectum, serotonergic neuron

## Abstract

Serotonin (5-hydroxytryptamine, 5-HT) is a phylogenetically conserved modulatory neurotransmitter. In mammals, 5-HT plays an important role in the regulation of many mental states and the processing of emotions in the central nervous system. Serotonergic neurons in the central nervous system, including the dorsal raphe (DR) and median raphe (MR) nuclei, are spatially clustered in the brainstem and provide ascending innervation to the entire forebrain and midbrain. Both between and within the DR and MR, these serotonergic neurons have different cellular characteristics, developmental origin, connectivity, physiology, and related behavioral functions. Recently, an understanding of the heterogeneity of the DR and MR serotonergic neurons has been developed at the molecular level. In birds, emotion-related behavior is suggested to be modulated by the 5-HT system. However, correspondence between the raphe nuclei of birds and mammals, as well as the cellular heterogeneity in the serotonergic neurons of birds are poorly understood. To further understand the heterogeneity of serotonergic neurons in birds, we performed a molecular dissection of the chick brainstem using *in situ* hybridization. In this study, we prepared RNA probes for chick orthologs of the following serotonin receptor genes: *5-HTR1A*, *5-HTR1B*, *5-HTR1D*, *5-HTR1E*, *5-HTR1F*, *5-HTR2A*, *5-HTR2B*, *5-HTR2C*, *5-HTR3A*, *5-HTR4*, *5-HTR5A*, and *5-HTR7.* We showed that the expression pattern of 5-HT receptors in the serotonin neurons of chick DR and MR may vary, suggesting heterogeneity among and within the serotonin neurons of the DR and MR in the chick brainstem. Our findings regarding the molecular properties of serotonergic neurons in the bird raphe system will facilitate a good understanding of the correspondence between bird and mammalian raphes.

## Introduction

Serotonin (5-hydroxytryptamine, 5-HT) is a phylogenetically conserved modulatory neurotransmitter (Marin et al., 2020). Many studies suggest that the influence of the 5-HT system on cognition, behavior, and emotion is evolutionarily conserved in the animal kingdom (Kandel et al., 2000; Bacque-Cazenave et al., 2020). To improve the understanding of the phylogenetic continuity of the neural basis for cognition and emotion, it is important to elucidate the neural circuits that process cognitive and emotional behaviors in animals other than mammals. Birds are model animals of interest for understanding the evolutionary continuity of the neural basis of cognition and emotion (Rosa Salva et al., 2015; Papini et al., 2019). It has been suggested that the 5-HT system controls aggressive behavior in pigeons (Fachinelli et al., 1989), feather pecking behavior (Kops et al., 2017; de Haas and van der Eijk, 2018) and foraging behavior in chickens (Matsunami et al., 2012). In addition, 5-HT transporter systems have been suggested to modulate fear-related behaviors in chickens using a combination of behavioral and functional polymorphisms (Krause et al., 2017, 2019; Phi Van et al., 2018). However, the neural circuits that are modulated by 5-HT and responsive 5-HT receptors (5-HTRs) in avian brains are largely unknown.

In mammals, the serotonergic neurons in the central nervous system are spatially clustered raphe nuclei in the brainstem, classically designated as groups B1–B9 (Dahlström and Fuxe, 1964; Soiza-Reilly and Gaspar, 2020). Among them, the dorsal raphe (DR: B6 and B7) and median raphe (MR: B5 and B8) are of special importance and have been intensively studied because they provide ascending innervation to the entire forebrain and midbrain (Olivier, 2015; Cohen and Grossman, 2020; Soiza-Reilly and Gaspar, 2020). A considerable heterogeneity of serotonergic neurons among and within DR and MR with respect to multiple aspects, such as developmental origin, connectivity, electrophysiological properties, and behavioral function exists (Calizo et al., 2011; Alonso et al., 2013; Okaty et al., 2019; Soiza-Reilly and Gaspar, 2020). The diversity of 5-HT neurons in the DR and MR has received particular attention in mammals (Okaty et al., 2019). Recently, the rapid development of single-cell RNA sequencing technology has provided a powerful tool for understanding the heterogeneity of serotonergic neurons at single-cell resolution among and within the DR and MR (Huang K. W. et al., 2019; Ren et al., 2019; Okaty et al., 2020). These studies have laid a molecular foundation for investigating the heterogeneity of serotonergic neurons in the DR and MR.

In contrast, the distribution and diversity of avian serotonergic neurons in the raphe are poorly understood currently. Only a few studies have reported the histological position of the avian raphe (Kuenzel and Masson, 1988; Puelles et al., 2007) and have investigated the distribution of 5-HT neurons in the avian brainstem, including the raphe. 5-HT neurons in the avian brainstem are divided into several groups based on histological features, such as the location, size, and shape and immunoreactivity (Yamada et al., 1984) or fluorescence chemical reactivity for the detection of monoamines (Ikeda and Gotoh, 1971; Dube and Parent, 1981). However, there are no studies on the distribution and diversity of 5-HTRs in the avian raphe. Furthermore, the correspondence between the mammalian and avian raphe is not yet clear.

In the present study, we investigated the chick brainstem at the molecular level using *in situ* hybridization (ISH) to improve the understanding of heterogeneity of 5-HT neurons in the avian raphe. In mammals, serotonergic neurons exhibit a high level of within-system communication *via* feedback where serotonin activates serotonin receptors on serotonergic neurons and a considerable heterogeneity of serotonin receptors exist among and within the serotonergic neurons of DR and MR. We selected chick orthologs of serotonergic neuron marker genes: tryptophan hydroxylase 2 (TPH2) and serotonin transporter (SERT), and the following serotonin receptor genes: *5-HTR1A*, *5-HTR1B*, *5-HTR1D*, *5-HTR1E*, *5-HTR1F*, *5-HTR2A*, *5-HTR2B*, *5-HTR2C*, *5-HTR3A*, *5-HTR4*, *5-HTR5A*, and *5-HTR7.* We found that the chick DR and MR serotonin neurons vary in the expression patterns of *5-HTR*s, suggesting heterogeneity of serotonin neurons among and within the DR and MR. Understanding the molecular properties of 5-HT neurons in the bird raphe will elucidate the correspondence between bird and mammalian raphes.

## Materials and Methods

### Animals

Fertilized eggs of domestic chicks of the Cobb strain (*Gallus gallus domesticus*) were purchased from a local dealer (3-M, Aichi, Japan) and incubated at the Teikyo University (Kaga, Itabashi-ku, Tokyo, Japan). Animal experiments were performed as described previously by Yamaguchi et al. (2008a; 2008b). Newly hatched chicks (P0) were transferred to dark plastic enclosures in a dark warm cage at 30°C for 1 day (P1). Previous studies showed that embryonic development of the serotonergic system is almost completed before hatching (Okado et al., 1992; Huang X. et al., 2019), suggesting that the distribution of serotonin neurons in the brainstem of P1 chick was almost same as that in the adult chick. We used P1 chick because we do not need to take care of rearing environment that might affect the level of gene expression. In this study, we used six chicks for tryptophan hydroxylase 2 (*TPH2)*, four for serotonin transporter (*SERT)*, four for the 5-*HTR1A* condition, two for the 5-*HTR1B*, five for the 5-*HTR1D*, four for the 5-*HTR1E*, two for the 5-*HTR1F*, one for the 5-*HTR2A*, one for the 5-*HTR2B*, one for the 5-*HTR2C*, one for the 5-*HTR3A*, one for the 5-*HTR4*, four for the 5-*HTR5A*, and two for the 5-*HTR7* (Supplementary Table 1). All the procedures were reviewed and approved by the Committee on Animal Experiments of Teikyo University and conducted in accordance with the guidelines of the national regulations for animal welfare in Japan.

### Histological Sample Preparations

P1 chicks were anesthetized by an intraperitoneal injection (0.40 mL/individual) of a 1:1 solution of ketamine (10 mg/mL, Ketalar-10, Sankyo Co., Tokyo, Japan) and xylazine (2 mg/mL, Sigma, St. Louis, MO, United States). The anesthetized chicks were transcardially perfused with 4% paraformaldehyde in 0.1 M phosphate buffered saline (pH 7.5, PFA-PBS). Whole brain specimens were dissected up to the spinal cord level and immediately immersed in PFA-PBS for 1 or 2 day(s) at 4°C and placed in an 18% sucrose/PFA-PBS solution for cryoprotection for 2 days at 4°C. Subsequently, brains with sucrose substitution were embedded in Tissue-Tek OCT compound (Sakura Finetechnical, Tokyo, Japan), frozen immediately on dry ice, and stored at −80°C until further use. The frozen brain blocks were cut into 18 μm-thick sections using a cryostat (Leica CM3050S or Leica CM1850, Leica Biosystems, Nußloch, Germany). The level of the serial coronal sections (A2.6 to A0.8) corresponded to those of the atlas by Kuenzel and Masson (1988).

### cDNA Cloning and RNA Probe Preparations

Total RNA was extracted from the chick brain using TRIzol Reagent (Invitrogen, Carlsbad, CA, United States) and reverse-transcribed using the SuperScript III kit (Invitrogen, Carlsbad, CA, United States) using an oligo (dT) primer, according to the manufacturer’s protocol. Reverse-transcription polymerase chain reaction (RT-PCR) was performed using the following gene-specific primer (forward and reverse) pairs: *TPH2:* 5′-TTTTGTGGACTGTGACTGCA-3′ and 5′-GATGCTCCCAATGAAGCGAG-3′, respectively; *SERT:* 5′-TACCCGCCAAGTTCTACAGG-3′ and 5′-CCTCCAA AAGTCAGGGTTGC-3′, respectively; 5-*HTR1D:* 5′-CCGTGG ATGAAAGGACACTG-3′ and 5′-TTCGTGAAGGCTTGCG TTAA-3′, respectively; 5-*HTR1E:* 5′-ACAACCCTGACT ATGCTGCT-3′ and 5′-AGCCAGGTCCAAGTCATTCT-3′, respectively; 5-*HTR1F:* 5′-TCCATTACCCTGTCTGTGCT-3′ and 5′-ATTCAGATCTGGGGCTTCGT-3′, respectively; 5-*HTR2B:* 5′-TGGAGCTGGCAATGGATCTT-3′ and 5′-CTTTTCTGGTGAGCAGGCAG-3′, respectively; 5-*HTR5A:* 5′-AGTAATCGGAGCGGGTCATC-3′ and 5′-CCGGACAGTGAAGACCATCT-3′, respectively; and 5-*HTR7:* 5′-CAACGGCAGCCACCTCTA-3′ and 5′-AAGAGCCACACACACAGGAT-3′, respectively. PCR products were subcloned into the pGEM-T easy vector (Promega, Madison, WI, United States). The sequences were confirmed using Sanger sequencing. For 5-*HTR1A, 5-HTR1B, 5-HTR2A, 5-HTR2C, 5-HTR3A*, and 5-*HTR4* probes, we used the plasmids which had already prepared in our previous study (Fujita et al., 2020). Plasmids containing the cDNA fragments for *TPH2, SERT, 5-HTR1A, 5-HTR1B, 5-HTR1D, 5-HTR1E, 5-HTR1F, 5-HTR2A, 5-HTR2B, 5-HTR2C, 5-HTR3A, 5-HTR4, 5-HTR5A*, and 5-*HTR7* were amplified by PCR using the M13 primer pair. Amplicons containing the T7 and SP6 promoter sites were purified using a PCR purification kit (Qiagen, Valencia, CA, United States). Digoxigenin (DIG)-labeled sense and antisense RNA probes were prepared by *in vitro* transcription using a DIG RNA labeling kit (Roche, Basel, Switzerland). For the double ISH analysis, *TPH2* fluorescein-labeled sense and antisense RNA probes were prepared by *in vitro* transcription using a fluorescein RNA labeling kit (Roche, Basel, Switzerland).

### *In situ* Hybridization

*In* s*itu h*ybridization was performed as described previously by Fujita et al. (2019), with some modifications. Specimens of brain sections were re-fixed in 4% PFA-PBS, pre-treated, and hybridized with DIG-labeled RNA probes at 70°C. After stringent washes, hybridized probes were detected *via* an immunohistochemical examination with an alkaline phosphatase-conjugated anti-DIG antibody (1:1,000; Roche, Basel, Switzerland). To visualize the signals, a chromogenic reaction with a nitro blue tetrazolium/5-bromo-4-chloro-3-indolyl phosphate (NBT/BCIP) was performed at room temperature (about 25°C) for the following durations: *TPH2* and *SERT*, 16–16.3 h; 5-*HTR1A*, 18–18.5 h; 5-*HTR1B*, 18.5–19.2 h; 5-*HTR1D*, 16.5–18 h; 5-*HTR5A*, 19.2 h; and 5-*HTR5A*, 18 h. Sense probes were used as negative controls for every experiment.

### Double *in situ* Hybridization

The process from re-fixation of sections to the chromogenic reaction with NBT/BCIP was performed as described above, except for hybridization. During hybridization, DIG-labeled RNA probes and fluorescein-labeled RNA probes were mixed and mounted simultaneously. After the first chromogenic reaction with NBT/BCIP, the anti-DIG antibody was detached using 100 mM glycine (pH 2.2). After washing in PBS, fluorescein-labeled probes were detected immunohistochemically with an alkaline phosphatase-conjugated anti-fluorescein antibody (1:1,000; Roche, Basel, Switzerland). To visualize the signals, the second color chromogenic reactions were performed at room temperature with SIGMAFAST Fast Red TR/Naphthol AS-MX tablets (Sigma-Aldrich, St. Louis, MO, United States cat#F4523) for the following durations: *5-HTR1A* and *TPH2*, 3 h and 18 h; *5-HTR1B* and *TPH2*, 3–16 h and 18–18.5 h; *5-HTR1D* and *TPH2*, 16 h and 19.3 h; *5-HTR1E* and *TPH2*, 2.5 h and 17.3 h; *5-HTR5A* and *TPH2*, 2.5 h and 17.3 h. Sense probes were used as negative controls for every experiment.

### Image Acquisition and Data Processing

Digital photographs of sections on each slide glass were acquired semi-automatically using NanoZoomer 2.0HT or NanoZoomer XR systems (Hamamatsu Photonics, Shizuoka, Japan). The microscopic fields of interest were cropped using NDP.view2 software (ver. 2.7.25^1^, Hamamatsu Photonics, Shizuoka, Japan). For the ISH images, the cropped images were converted to 8-bit images, and their brightness and contrast were adjusted using ImageJ (ver. 1.52a, National Institute of Health, United States^2^).

### Terminology of the Bird Raphe

All the histological terminologies used in this study followed the atlas of Kuenzel and Masson (1988) and the description of the avian brain nomenclature consortium (Reiner et al., 2004), except for the histological position of the raphe and the terminologies of serotonergic neuron cell groups. The histological position of the raphe follows the nomenclature by Puelles et al. (2007). As for serotonergic neuron cell groups, several studies determined the distribution of chicken serotonergic neuron cell groups and proposed possible correspondence to mammalian groups (Ikeda and Gotoh, 1971; Dube and Parent, 1981; Yamada et al., 1984). Based on previous studies (Ikeda and Gotoh, 1971; Yamada et al., 1984; Puelles et al., 2007), we used the terms DR and MR for the serotonergic neuron cell groups. The relationship between the terminologies used in this study and that in previous studies are summarized in Table 1.

**TABLE 1:** Comparison of the terminology regarding the serotonergic neuron cell groups and possible correspondence between birds and mammals.

Birds (chicken)				Mammals (rat)
This study, 2021	Ikeda and Gotoh, 1971	Yamada et al., 1984	Puelles et al., 2007	Dahlström and Fuxe, 1964
Dorsal raphe	B5	Group 5	DR	B6
				B7
Median raphe	B6	Group 6	r1MnR	B5
			r2MnR	
	B7	Group 10	CLi	B8

*CLi, caudal linear nucleus of the raphe; DR, dorsal raphe nucleus; r1MnR, rhombomere 1 median raphe nucleus; r2MnR, rhombomere 2 median raphe nucleus.*

## Results

### Selection of the Chick Orthologs of Mammalian Serotonergic Neuron Marker Genes and 5-Hydroxytryptamine Receptor Genes

We selected chick orthologs of the following mammalian serotonergic neuron marker genes: *Tph2*, *Sert*, and all the chick *5-HTR* genes (*5-HTR1A, 5-HTR1B, 5-HTR1D, 5-HTR1E, 5-HTR1F, 5-HTR2A, 5-HTR2B, 5-HTR2C, 5-HTR3A, 5-HTR4, 5-HTR5A*, and *5-HTR7*; except *5-HTR6*, for which we could not obtain a subclone). TPH2 is a key enzyme for serotonin biosynthesis (Walther and Bader, 2003; Walther et al., 2003). SERT is a transporter responsible for serotonin uptake from the synaptic cleft to the presynaptic neuron and is encoded by the solute carrier family 6 member 4 (*Slc6a4*) gene (Hoffman et al., 1991; Lesch et al., 1993). The orthologs exhibited the following sequence similarities (protein and DNA) between chicks and humans: *TPH2*, 91.6% and 81.1%, respectively; *SERT*, 81.9% and 76.9%, respectively; *5-HTR1D*, 79.8% and 71.6%, respectively; *5-HTR1E*, 86.8% and 75.7%, respectively; *5-HTR1F*, 83.2% and 79.1%, respectively; *5-HTR2B*, 79.5% and 73.9%, respectively; *5-HTR5A*, 80% and 77.6%, respectively; and *5-HTR7*, 84.6% and 79.2%, respectively. We searched for sequence similarities of *5-HTR1A, 5-HTR1B, 5-HTR2A, 5-HTR2C, 5-HTR3A*, and *5-HTR4* with other animals in a previous study (Fujita et al., 2020). The characteristics of these ortholog gene probes are summarized in Table 2. We designed probes to detect multiple transcript variants of the orthologs registered in the database. We performed ISH and analyzed the expression patterns of orthologs in the brainstem of chicks.

**TABLE 2:** Overview of the serotonergic neuron marker and *5-HTR*s probes used in this study.

Accession number	Gene symbol	Molecular characteristics	Probe preparation
NM_001001301.1	*TPH2*	Enzyme	This study
NM_213572.1	*SERT*	Membrane transporter	This study
NM_001170528.1	*5-HTR1A*	GPCR	Fujita et al., 2020
XM_015284634.2	*5-HTR1B*	GPCR	Fujita et al., 2020
XM_015297583.1	*5-HTR1D*	GPCR	This study
XM_015284709.2	*5-HTR1E*	GPCR	This study
XM_004938334.3	*5-HTR1F*	GPCR	This study
XM_025151250.1	*5-HTR2A*	GPCR	Fujita et al., 2020
XM_025153310.1	*5-HTR2B*	GPCR	This study
XM_004940651.3	*5-HTR2C*	GPCR	Fujita et al., 2020
XM_004948063.3	*5-HTR3A*	Ligand-gated ion channel	Fujita et al., 2020
XM_015293658.2	*5-HTR4*	GPCR	Fujita et al., 2020
XM_425970.3	*5-HTR5A*	GPCR	This study
XM_015288394.2	*5-HTR7*	GPCR	This study

*5-HTR, 5-hydroxytryptamine receptor; GPCR, G protein-coupled receptor; SERT, serotonin transporter; TPH, tryptophan hydroxylase 2.*

### *TPH2* and *SERT* Expression in the Chick Brainstem

**FIGURE 1:**
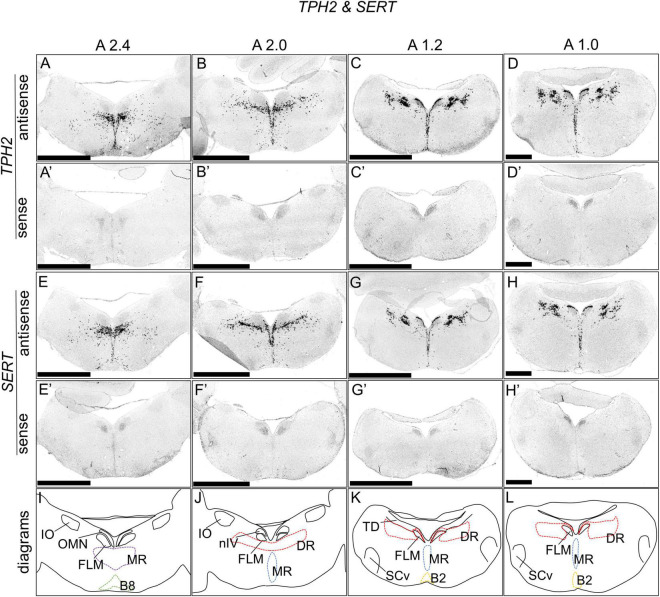
*In situ* hybridization of *TPH2* and *SERT* in the P1 chick brainstems. Digoxigenin-labeled RNA antisense [*TPH2* **(A–D)**, and *SERT* **(E–H)**] and sense [*TPH2* **(A’–D’)** and *SERT* **(E’–H’)**] probes were used for *in situ* hybridization in P1 chick brain coronal sections. To evaluate the expression patterns of *TPH2* and *SERT*, sections of six chicks for *TPH2* and four chicks for *SERT* were analyzed, and the representative levels of sections (A2.4, A2.0, A1.2, and 1.0) are shown. The levels of the sections are in accordance with those mentioned in Kuenzel and Masson’s chick atlas (Kuenzel and Masson, 1988). **(I–L)** Diagrams of coronal sections shown in panels **(A–D)**. B2, B2 cell group; B8, B8 cell group; DR, dorsal raphe; FLM, fasciculus longitudinalis medialis; IO, nucleus isthmo-opticus; MR, median raphe; nIV, nucleus nervi trochlearis; OMN, oculomotor nucleus; SCv, nucleus suboeruleus ventralis; P1, post-hatch day 1. Scale bars = 2.5 mm **(A–C,A’–C’)** and 1 mm **(D,D’,H,H’)**.

We performed an ISH analysis to reveal the expression patterns of the serotonergic neuron markers *TPH2* and *SERT* with neighbor sections around A2.4 to A 1.0 in the brainstems of naive chicks on post-hatch day 1 (P1). We detected cells showing strong signals of *TPH2* and *SERT* in the MR (B7 cell cluster) and B8 cell cluster (Figures 1A,E,A’,E’), DR (B5 cell cluster), and MR (B6 cell cluster, Figures 1B–D,F–H,B’–D’,F’–H’), and B2 cell clusters (Figures 1C,D,G,H,C’,D’,G’,H’), respectively. Serotonergic neurons are widely distributed in the MR and DR (Dahlström and Fuxe, 1964; Soiza-Reilly and Gaspar, 2020). The distribution of serotonergic neurons in chick brainstems with *TPH2* and *SERT* signals was similar (Ikeda and Gotoh, 1971; Dube and Parent, 1981; Yamada et al., 1984), showing that these two chick ortholog genes are useful markers for visualizing serotonergic neurons in the avian brainstem.

### *5-HTR1A* Expression in the Chick Brainstem

**FIGURE 2:**
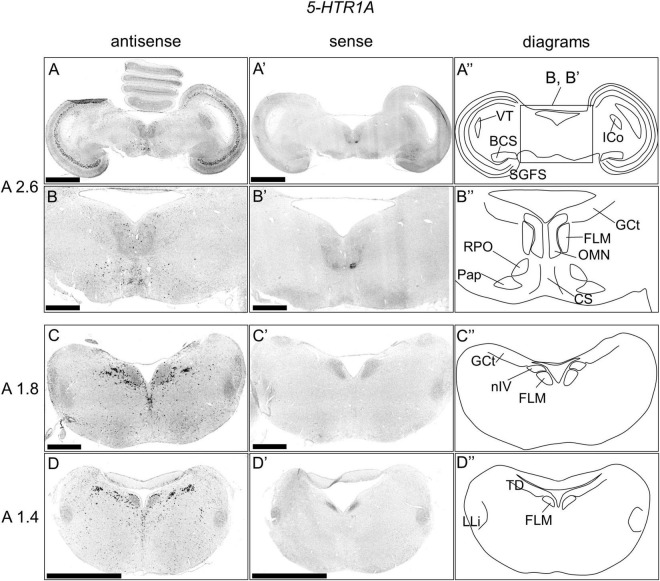
*In situ* hybridization of *5-HTR1A* in the P1 chick brainstems. Digoxigenin-labeled RNA antisense **(A–D)** and sense **(A’–D’)** *5-HTR1A* probes were used for *in situ* hybridization in coronal sections of P1 chick brains. To evaluate the expression patterns of *5-HTR1A*, sections of four chicks were analyzed, and representative images of two chick brain sections are shown. **(B,B’)** Magnified views of brain areas in the box in panel **(A”)** are shown. **(A”–D”)** Diagrams of coronal sections are shown in the rightmost panels. The levels of the sections (A 2.6, A 1.8, and A 1.4) are in accordance with those mentioned in Kuenzel and Masson’s chick atlas (Kuenzel and Masson, 1988). BCS, brachium colliculi superiors; CS, nucleus centralis superior; FLM, fasciculus longitudinalis medialis; GCt, griseum centrale; ICo, nucleus intercolicularis; LLi, nucleus ventralis lemnisci lateralis; nIV, nucleus nervi trochlearis; OMN, oculomotor nucleus; Pap, nucleus papillioformis; RPO, nucleus reticularis pontis oralis; SGC, stratum griseum centrale; TD, nucleus tegmenti dorsalis; VT, ventriculus tecti mesencephali; P1, post-hatch day 1. Scale bars = 2.5 mm **(A,D,A’,D’)** and 1 mm **(B,C,B’,C’)**.

We examined the expression pattern of 5-*HTR1A* in sections A2.6 to A1.4 in the P1 chick brainstems (Figure 2). Strong signals were detected in a layer of the stratum griseum centrale (SGC; Figures 2A,A’). We detected cells showing strong signals in the nucleus intercolicularis (ICo; Figures 2A,A’), nucleus centralis superior (CS), fasciculus longitudinalis medialis (FLM), oculomotor nucleus (OMN), nucleus papillioformis (Pap), and nucleus reticularis pontis oralis (RPO; Figures 2B,B’). In addition, we detected cells with strong signals in the B5 cell cluster and B6 cell cluster and cells with relatively weak signals in other areas (Figures 2C,D,C’,D’).

### *5-HTR1B* Expression in the Chick Brainstem

**FIGURE 3:**
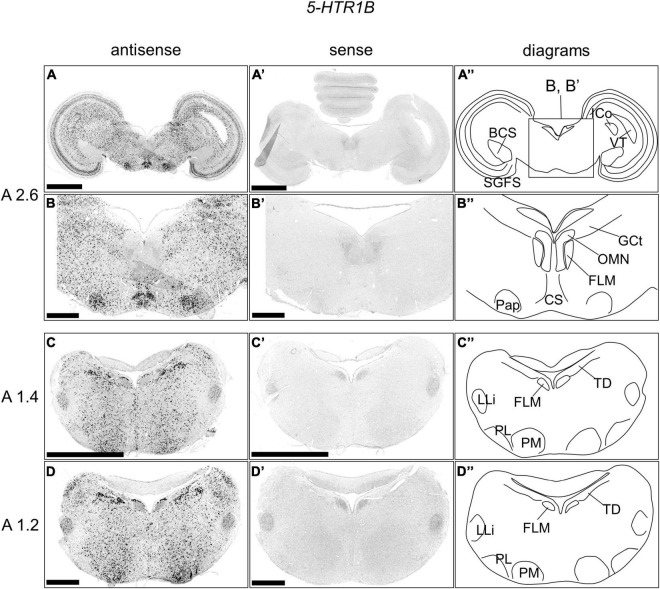
*In situ* hybridization of *5-HTR1B* in the P1 chick brainstems. Digoxigenin-labeled RNA antisense **(A–D)** and sense **(A’–D’)** *5-HTR1B* probes were used for *in situ* hybridization in coronal sections of P1 chick brains. To evaluate the expression patterns of *5-HTR1B*, sections of two chicks were analyzed, and representative images of chick brain sections are shown. **(B,B’)** Magnified views of brain areas in the box in panel **(A”)** are shown. **(A”–D”)** Diagrams of coronal sections are shown in the rightmost panels. The levels of the sections (A 2.6, A 1.4, and A 1.2) are in accordance with those mentioned in Kuenzel and Masson’s chick atlas (Kuenzel and Masson, 1988). BCS, brachium colliculi superiors; CS, nucleus centralis superior; FLM, fasciculus longitudinalis medialis; GCt, griseum centrale; ICo, nucleus intercolicularis; LLi, nucleus ventralis lemnisci lateralis; OMN, oculomotor nucleus; Pap, nucleus papillioformis; PL, nucleus pontis lateralis; PM, nucleus pontis medialis; SGFS, stratum griseum et fibrosum superficiale; SGP, stratum griseum periventriculare; TD, nucleus tegmenti dorsalis; VT, ventriculus tecti mesencephali; P1, post-hatch day 1. Scale bars = 2.5 mm **(A,C,A’,C’)** and 1 mm **(B,D,B’,D’)**.

We examined the expression pattern of 5-*HTR1B* in sections A2.6 to A1.2 in the P1 chick brainstems (Figure 3). Strong signals were detected in a wide area of the Optic tectum (OT), except for the brachium colliculi superiors, *5-HTR1A* positive layers, and ICo (Figures 3A,A’). We also detected strong signals in a wide area of the brainstem, including the Pap, except for the FLM and OLM (Figures 3B,B’). In addition, we detected cells with strong signals in a wide area of the brainstem, including the B5 cell cluster, B6 cell cluster, nucleus pontis medialis, and nucleus ventralis lemnisci lateralis (LLi), except for the FLM and nucleus pontis lateralis and around the LLi (Figures 3C,D,C’,D’).

### Comparison of the ***5-HTR1A*** and ***5-HTR1B*** Expression Patterns in the OT

**FIGURE 4:**
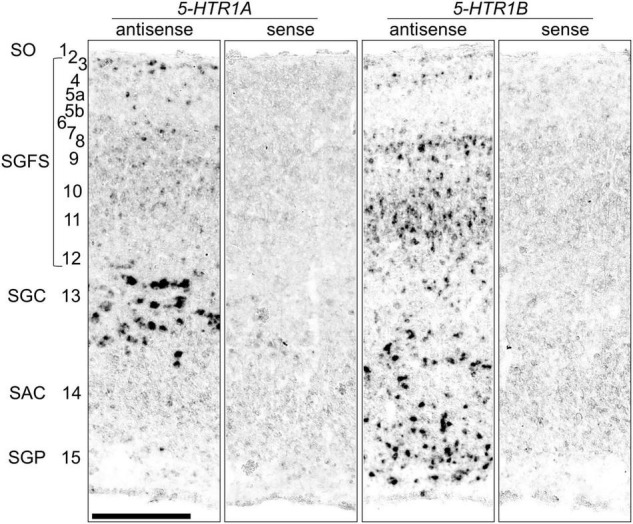
Comparison of the *5-HTR1A* and *5-HTR1B* expression patterns in the optic tectum in the P1 chick brainstem sections. Magnified views of the regions of the optic tectum to compare layers of *5-HTR1A*-expressing and *5-HTR1B*-expressing areas. Numbers represent tectal layers according to Ramón y Cajal (1911). The leftmost alphabetical system of nomenclature is in accordance with that mentioned in Kuenzel and Masson’s chick atlas (Kuenzel and Masson, 1988). SAC, stratum album centrale; SGC, stratum griseum centrale; SGFS, stratum griseum et fibrosum superficiale; SGP, stratum griseum periventriculare; SO, stratum opticum; P1, post-hatch day 1. Scale bars = 250 μm.

To compare the expression patterns of *5-HTR1A* and *5-HTR1B*, we show a magnified view of the OT (Figure 4). We detected strong *5-HTR1A* signals in the SGC (layer 13) and sparse signals in the stratum griseum et fibrosum superficiale (SGFS; layers 2, 3, 5, and 7). On the contrary, we detected strong *5-HTR1B* signals in the stratum album centrale (layer 14), stratum griseum periventriculare (layer 15), and SGFS (layers 4, 8, 10, 11, and 12). The expression patterns of *5-HTR1A* and *5-HTR1B* in the OT layers were mutually exclusive.

### *5-HTR1D* Expression in the Chick Brainstem

**FIGURE 5:**
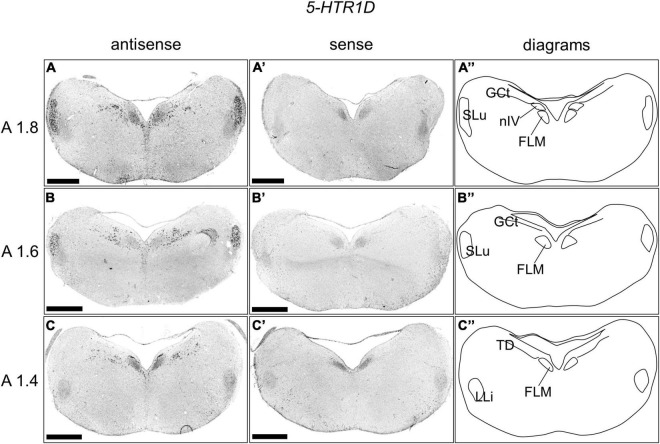
*In situ* hybridization of *5-HTR1D* in the P1 chick brainstems. Digoxigenin-labeled RNA antisense **(A–C)** and sense **(A’–C’)** *5-HTR1D* probes were used for *in situ* hybridization in coronal sections of P1 chick brains. To evaluate the expression patterns of *5-HTR1D*, sections of five chicks were analyzed, and representative images of two chick brain sections are shown. **(A”–C”)** Diagrams of coronal sections are shown in the rightmost panels. The levels of the sections (A1.8, A1.6, and A1.4) are in accordance with those mentioned in Kuenzel and Masson’s chick atlas (Kuenzel and Masson, 1988). FLM, fasciculus longitudinalis medialis; GCt, griseum centrale; LLi, nucleus ventralis lemnisci lateralis; nIV, nucleus nervi trochlearis; SLu, nucleus semilunaris; TD, nucleus tegmenti dorsalis; P1, post-hatch day 1. Scale bars = 1 mm.

We examined the expression level of 5-*HTR1D* in sections A1.8 to A1.4 in the P1 chick brainstem (Figure 5). Strong signals were detected in the nucleus semilunaris (SLu; Figures 5A,B,A’,B’). In addition, cells with strong signals were distributed in the DR (B5 cell cluster), and cells with relatively weak signals were distributed in the MR (B6 cell cluster; Figures 5A–C,A’–C’).

### *5-HTR1E* Expression in the Chick Brainstem

**FIGURE 6:**
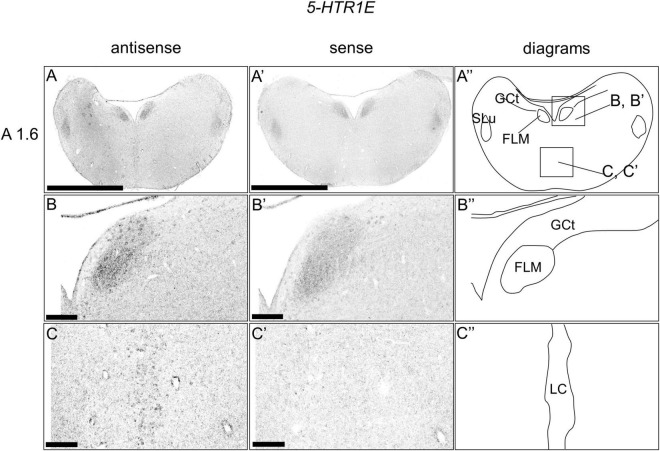
*In situ* hybridization of *5-HTR1E* in the P1 chick brainstems. Digoxigenin-labeled RNA antisense **(A–C)** and sense **(A’–C’)** *5-HTR1E* probes were used for *in situ* hybridization in coronal sections of P1 chick brains. To evaluate the expression patterns of *5-HTR1E*, sections of four chicks were analyzed, and the representative levels of sections (A 1.6) are shown. **(A”–C”)** Diagrams of coronal sections shown in the rightmost panels. The levels of the sections (A 1.6) is in accordance with those mentioned in Kuenzel and Masson’s chick atlas (Kuenzel and Masson, 1988). **(B–C,B’–C’)** Magnified views of brain areas shown in the boxes in panel **(A”)**. FLM, fasciculus longitudinalis medialis; GCt, griseum centrale; MR, median raphe; SLu, nucleus semilunaris; P1, post-hatch day 1. Scale bars = 2.5 mm **(A,A’)** and 250 μm **(B–C,B’–C’)**.

We examined the expression level of *5-HTR1E* in the sections of the brainstem and did not detect clear expression patterns (Figures 6A,A’,A”). However, when we observed the sections in detail, we detected cells with relatively weak signals in a part of the DR (B5 cell cluster; Figures 6B,B’,B”) and MR (B6 cell cluster; Figures 6C,C’,C”), respectively.

### *5-HTR5A* Expression in the Chick Brainstem

**FIGURE 7:**
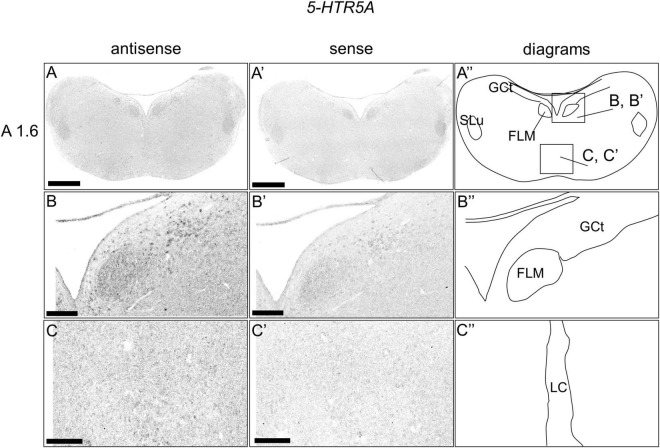
*In situ* hybridization of *5-HTR5A* in the P1 chick brainstems. Digoxigenin-labeled RNA antisense **(A–C)** and sense **(A’–C’)** *5-HTR5A* probes were used for *in situ* hybridization in coronal sections of P1 chick brains. To evaluate the expression patterns of *5-HTR5A*, sections of four chicks were analyzed, and the representative levels of sections (A1.6) are shown. **(A”–C”)** Diagrams of coronal sections shown in the rightmost panels. The levels of the sections (A 1.6) is in accordance with those mentioned in Kuenzel and Masson’s chick atlas (Kuenzel and Masson, 1988). **(B–C,B’–C’)** Magnified views of brain areas shown in the boxes in panel **(A”)**. FLM, fasciculus longitudinalis medialis; GCt, griseum centrale; MR, median raphe; SLu, nucleus semilunaris; P1, post-hatch day. Scale bars = 1 mm **(A,A’)** and 250 μm **(B–C,B’–C’)**.

We examined the expression level of *5-HTR5A* in the brainstem sections, and we did not detect clear expression patterns when the entire sections of brainstem were observed (Figures 7A,A’,A”). When we observed the sections in detail, we detected cells with relatively weak signals in a part of the DR (B5 cell cluster; Figures 7B,B’,B”) and MR (B6 cell cluster; Figures 7C,C’,C”).

### *5-HTR1F, 5-HTR2A, 5-HTR2B, 5-HTR2C, 5-HTR3A, 5-HTR4*, and *5-HTR7* Expression Patterns in the Chick Brainstem

We examined *5-HTR1F, 5-HTR2A, 5-HTR2B, 5-HTR2C, 5-HTR3A, 5-HTR4*, and *5-HTR7* expression levels in sections around A2.4 to A0.8 in the P1 chick brainstem and did not detect any signal, suggesting that the expression levels of the *5-HTR* genes were very low or the cells expressing the *5-HTR* genes were very rare. In mammals, the 5-HT2B, 2C or 5-HT7 receptors can control the activity of raphe neurons (Beyeler et al., 2021) but their expression level is very low (Huang K. W. et al., 2019; Okaty et al., 2019; Ren et al., 2019). This is consistent with our results that the expression level of the 5-HT2B, 2C or 5-HT7 receptors was too low to detect by our *in situ* hybridization system in this study.

### Double *in situ* Hybridization Analysis of the 5-Hydroxytryptamine Receptor and Serotonergic Neuron Marker Genes in the Chick Brainstem

**FIGURE 8:**
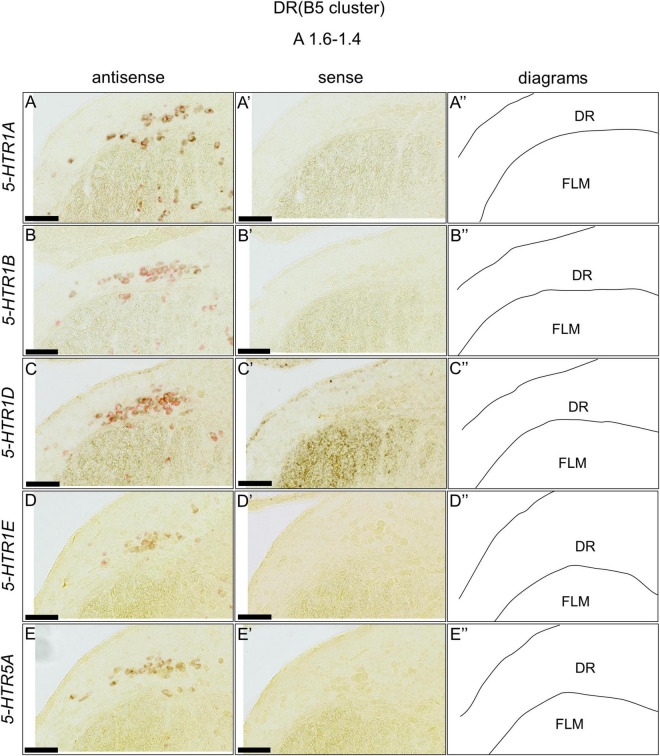
Double *in situ* hybridization of *5-HTRs* and *TPH2* in the DR (B5) of the P1 chick brainstems. Digoxigenin-labeled (*5-HTRs*) and fluorescein-labeled *TPH2* RNA antisense **(A–E)** and sense **(A’–E’)** probes were used for double *in situ* hybridization in coronal sections of P1 chick brains. **(A”–E”)** Diagrams of coronal sections are shown in the rightmost panels. DR, dorsal raphe; FLM, fasciculus longitudinalis medialis; P1, post-hatch day 1. Scale bars = 100 μm.

**FIGURE 9:**
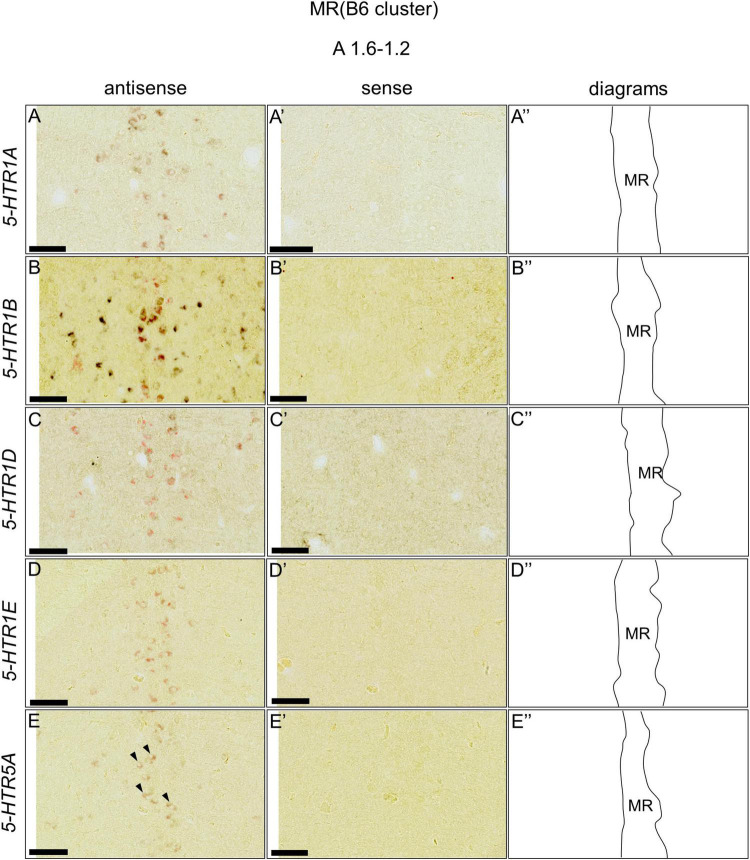
Double *in situ* hybridization of *5-HTRs* and *TPH2* in the MR (B6) of the P1 chick brainstems. Digoxigenin-labeled (*5-HTRs*) and fluorescein-labeled *TPH2* RNA antisense **(A–E)** and sense **(A’–E’)** probes were used for double *in situ* hybridization in coronal sections of P1 chick brains. **(A”–E”)** Diagrams of coronal sections are shown in the rightmost panels. Arrowheads indicate signals. MR, median raphe; P1, post-hatch day 1. Scale bars = 100 μm.

We found that five 5-HTRs (*5-HTR1A, 5-HTR1B, 5-HTR1D, 5-HTR1E*, and *5-HTR5A*) were expressed in the DR (B5 cell cluster) and MR (B6 cell cluster), as mentioned above. Subsequently, we examined whether the *5-HTR*-expressing cells were serotonergic neurons. We performed a double ISH analysis to reveal that the *5-HTR*-expressing cells expressed a serotonergic neuron marker gene, *TPH2*, at the section level around A1.6 to A1.4 for the DR (B5 cell cluster) and A1.6 to A 1.2 for the MR (B6 cell cluster; Figures 8, 9). We detected double positive cells of *5-HTR1A* and *TPH2*, *5-HTR1B* and *TPH2*, and *5-HTR1D* and *TPH2*, and a small number of double positive cells of *5-HTR1E* and *TPH2;* and *5-HTR5A* and *TPH2* in the DR (B5 cell cluster; Figures 8A–E,A’–E’). In the B6 cell cluster of the MR, we detected double positive cells of *5-HTR1A* and *TPH2*, *5-HTR1B* and *TPH2*, *5-HTR1E* and *TPH2*. We also detected double-positive cells of *5-HTR1D* and in a part of the MR (B6 cell cluster), and weakly double-positive cells of *5-HTR5A* and *TPH2* in a part of the MR (B6 cell cluster; Figures 9A–E,A’–E’).

## Discussion

### Distribution of Serotonergic Neurons in the Brainstem of Chicks

In the present study, we described the distribution of serotonergic neurons in the brainstem of chicks, which expressed the chick orthologs of mammalian serotonergic neuronal marker genes *TPH2* and *SERT*, including the chick DR and MR (Figure 1). The expression patterns of *TPH2* and *SERT* in the avian brainstem appears to correspond the distribution of serotonergic neurons in chick brainstems (Ikeda and Gotoh, 1971; Dube and Parent, 1981; Yamada et al., 1984), showing that these two chick ortholog genes are useful markers for visualizing serotonergic neurons in the avian brainstem. Our ISH analysis shows that serotonergic neurons are distributed laterally in the avian brainstem, especially in the DR, which is consistent with the finding of previous studies (Ikeda and Gotoh, 1971; Dube and Parent, 1981; Yamada et al., 1984; Wallace, 1985; Sako et al., 1986; Okado et al., 1992; Huang X. et al., 2019). In previous studies, chick serotonergic neurons in the brainstem were detected using histochemical (Ikeda and Gotoh, 1971; Dube and Parent, 1981) or immunohistochemical methods with anti-5-HT antiserum (Yamada et al., 1984); however, the authors did not clearly detect the distribution of serotonergic neuronal cell bodies alone because of the limitations of their methods. Generally, cell bodies can be clearly detected using ISH. In the future, a good understanding of the strict correspondence between the raphe nuclei of birds and mammals should be elucidated by a comprehensive analysis of the expression patterns of *TPH2* and *SERT* in the bird brainstem.

### Heterogeneity of Chick Serotonergic Neurons in the Chick Brainstem

**FIGURE 10:**
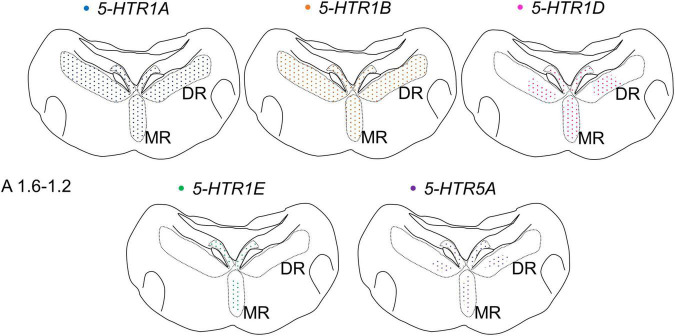
Schematic summary of the expression patterns of *5-HTR1A, 5-HTR1B, 5-HTR1D, 5-HTR1E*, and *5-HTR5A* in the DR and MR in the P1 chick brainstems. Representative expression patterns in sections around A 1.6. A 1.2 are exhibited in colored areas (blue, *5-HTR1A*; orange, *5-HTR1B*; magenta, *5-HTR1D*; green, *5-HTR1E*; purple, *5-HTR5A*). The dotted pattern indicates that the expressed cells are sparsely distributed. The levels of the sections are in accordance with those mentioned in Kuenzel and Masson’s chick atlas (Kuenzel and Masson, 1988). DR, dorsal raphe; MR, median raphe; P1, post-hatch day 1.

We also found that the expression patterns of *5-HTR1A, 5-HTR1B, 5-HTR1D, 5-HTR1E*, and *5-HTR5A* were different but overlapped partially in the DR and MR of the chick brainstem (Figure 10). Cells expressing *5-HTR1A* and/or *5-HTR1B* were widely distributed in the DR; however, the number of cells that expressed *5-HTR1D* or *5-HTR5A* in the DR was limited. Cells expressing *5-HTR1E* were not detected in the DR, except in the dorsal region of the FLM. In the MR, cells expressing *5-HTR1A* and/or *5-HTR1B* and/or *5-HTR1D* were widely distributed but those expressing *5-HTR1E* or *5-HTR5A* were limited. Moreover, cells expressing 5-HTRs also expressed the serotonergic neuronal marker gene, *TPH2*, at least partially (Figures 8, 9). Taken together, the serotonergic neurons in the chick DR and MR vary in their expression pattern of 5-HTRs, suggesting a heterogeneity of serotonergic neurons among and within the DR and MR in the chick brainstem. On the contrary, in mammals, single-cell RNA sequencing studies recently revealed that *5-Htr1a 5-Htr1b*, and *5-Htr1d* are widely expressed in the DR and MR of the mouse brainstem; however, *5-Htr5b* is expressed in limited clusters of the DR and MR. Given the absence of *5-Htr1e* in the mouse genome and the absence of *5-HTR5b* in the chicken genome (International Chicken Genome Sequencing Consortium, 2004; Vilaró et al., 2020), it seems likely that the *5-HTR* subtypes expressed in the DR and MR serotonergic neurons are similar in chicks and mice. Collectively, we assume that the molecular properties of serotonergic neurons present in the DR and MR of birds and mammals are similar in terms of *5-HTR* subtypes, raising the possibility that with a few exceptions, the heterogeneity of serotonergic neurons is evolutionarily conserved. Our assumption is consistent with the previous reports about the development of the brainstem and the pattern of gene expression in chicks, which resembles that of mice, even though the species are separated by approximately 300 million years of evolution (Cambronero and Puelles, 2000; Alonso et al., 2013; Watson et al., 2019).

### Expression Patterns of *5-HTR1A* and *5-HTR1B* in the OT Layers

During the process of detecting the expression of 5-*HTR*s in raphe, we happened to find that the expression patterns of *5-HTR1A* and *5-HTR1B* in the OT layers were mutually exclusive (Figures 2A,A’, 3A,A’, 4). The avian brain has extensively developed optic lobes and a high degree of lamination. The OT is a component of the tectofugal pathway, comprising the retina, OT, nucleus rotundus, and entopallium, which is thought to be the most important visual pathway in birds (Wylie et al., 2009). In the OT, there are 15 recognizable layers and a strict separation of visual input and output; generally, the input from the retina terminates in the superficial layer, while the major output from the OT to the nucleus rotundus is derived from the projection neurons in the deep layer, especially layer 13 (SGC) (Ramón y Cajal, 1911; Luksch, 2003). The cells expressing *5-HTR1A* were distributed mainly in layer 13 and sparsely in layers 2, 3, and 5 (Figure 4). Conversely, cells expressing *5-HTR1B* were not distributed in layer 13 and existed in layers 4, 8, 10, 11, 12, 14, and 15 (Figure 4). However, a previous study using radioligand autoradiography revealed that the density of 5-HTR1A densities was the strongest in layer 5 and decreased stepwise until layer 14 in the OT of pigeons (Herold et al., 2012). The difference in the 5-HTR1A signal density among different layers may reflect the density of the axonal terminals of 5-HT neurons projecting to the OT. Moreover, it is known that serotonergic neuron projections to the OT are widely distributed (Yamada and Sano, 1985). Collectively, *5-HTR1A* and *5-HTR1B*, which have characteristic expression patterns in the OT, might play important roles in the modulation of neurons, including visual processing in the tectofugal pathway. Note that we did not perform a comprehensive search for the expression of 5-HTR subtypes in the avian OT. In this study, we focused on the expression of *5-HTR*s in raphe rather than in the OT. In fact, a previous study that used immunohistochemistry reported that 5-HTR2A was strongly expressed in layer 13 cells and was widely detected in the OT (Metzger et al., 2006). This must be addressed in the future through a comprehensive search for the expression of 5-HTR subtypes in the avian OT.

### Possible Functions of Chick 5-Hydroxytryptamine Receptors in Serotonergic Neurons

As for the expression pattern of *5-HTR1A*, cells expressing *5-HTR1A* were distributed densely in the DR and MR and sparsely in the FLM, OMN, Pap, and RPO of the chick brainstem. We found that the distribution pattern of cells expressing *5-HTR1A* seemed to represent the overall distribution of serotonergic neurons, suggesting that *5-HTR1A* is expressed in most of the serotonergic neurons in the bird raphe. This is consistent with the findings of previous reports on the raphe in rodents, which showed that *5-Htr1A* is expressed in all serotonergic neurons (Day et al., 2004; Bonnavion et al., 2010; Vilaró et al., 2020). In mammals, 5-Htr1A is the major receptor involved in feedback inhibition of serotonergic neurons *via* direct and indirect (multisynaptic) feedback inhibition (Commons, 2020). The function of 5-HTR1A in serotonergic neuron feedback systems may be evolutionarily conserved in birds and mammals.

As for the expression pattern of *5-HTR1B*, cells expressing *5-HTR1B* were distributed widely in the brainstem of chicks, including the DR and MR, except for the FLM and OLM. Consistent with our results regarding the expression pattern of *5-HTR1B* in the brainstem of chicks, previous studies using mammals revealed that *5-Htr1B* is expressed widely, including the raphe (Bruinvels et al., 1994; Varnäs et al., 2005). In mammals, the localization of the 5-Htr1B receptor protein does not frequently correspond to that of the mRNA in the brain areas, suggesting that 5-Htr1B also functions as presynaptic auto- or heteroreceptors and modulates the release of various neurotransmitters (Vilaró et al., 2020). The presynaptic feedback mechanisms mediated by 5-Htr1B, located on serotonergic boutons, are expected to finely regulate local axonal excitation in a synapse-specific manner (Commons, 2020). Considering the similarity of expression patterns between the raphe of birds and mammals, we assumed that the modulatory function of 5-HTR1B in serotonergic neurons may be conserved among birds and mammals.

*5-HTR1D* was densely expressed in the SLu, which is one of the isthmic nuclei in birds and has reciprocal connections with the ipsilateral OT (Luksch, 2003). It is possible that *5-HTR1D* is also related to the regulation of visual function in birds. We also found that *5-HTR1D* was expressed in serotonergic neurons in the DR and MR of chicks. In terms of the mode of action in mammals, *5-Htr1D* is considered to act as an auto- or heteroreceptor and modulate the release of various neurotransmitters, since protein and mRNA localization does not correspond in some brain regions (Bruinvels et al., 1994). Bird *5-HTR1D* may act as an auto- or heteroreceptor for serotonergic neurons and might be involved in the regulation of the serotonergic system in birds.

As for the expression pattern of *5-HTR1E*, cells expressing *5-HTR1E* were serotonergic neurons, and they were distributed in the DR and MR. In mammals, the distribution of *5-ht1e* has been described in guinea pigs, monkeys, and the human brain (Bruinvels et al., 1994; Mengod et al., 2006) and by using antibodies against the 5-ht1e receptor in guinea pigs (Klein and Teitler, 2012). However, previous studies have not clearly demonstrated the expression of *5-ht1e* in the raphe nuclei. Since the *5-Htr1E* gene is absent in the genomes of both rats and mice, there is no previous study on the distribution of *5-Htr1E* in these animals even though they are the most common model animals. Here, we clearly showed that *5-Htr1E* was expressed in serotonergic neurons in the DR and MR in the chick brainstem. In the serotonergic neurons of the DR and MR in birds, *5-HTR1E* can be involved in the regulatory function of the serotonergic system.

Further, cells expressing *5-HTR5A* were distributed in the DR and MR and co-expressed the serotonergic neuron marker *TPH2*. The function of *5-HTR5A* has not been well characterized, even in mammals (Vilaró et al., 2020); however, there are a few studies on the distribution of *5-HTR5A* in mammalian brains (Plassat et al., 1992; Erlander et al., 1993; Matthes et al., 1993; Kinsey et al., 2001; Mengod et al., 2006). The distribution patterns suggested that *5-HTR5A* might be involved in the regulation of cognition, anxiety, and sensory perception. In addition, immunohistochemistry showed that *5-HTR5A* is localized in serotonergic neurons in the raphe nuclei of the rat brain (Oliver et al., 2000), suggesting that *5-Htr5A* might be an auto- or heteroreceptor that responds to a neurotransmitter released from the serotonergic neurons. Considering the similarity of expression patterns in raphe nuclei among birds and mammals, we assume that *5-HTR5A* might have a conserved modulatory function as an auto- or heteroreceptor in serotonergic neurons of birds and mammals.

## Conclusion

We have comprehensively described the serotonergic neurons in the DR and MR of the chick brainstem and found that *5-HTR1A, 5-HTR1B, 5-HTR1D, 5-HTR1E*, and *5-HTR5A* were expressed in serotonergic neurons. These receptors may be involved in the regulation of bird serotonergic neuron activity to control the serotonergic system. In addition, we found that *5-HTR1A* and *5-HTR1B* were expressed in some layers of the OT in a mutually exclusive manner. Our findings can be used as a basis for understanding the serotonergic modulation of serotonergic neurons themselves and the visual pathway in birds.

## Data Availability Statement

The raw data supporting the conclusions of this article will be made available by the authors, without undue reservation.

## Ethics Statement

The animal study was reviewed and approved by the Committee on Animal Experiments of Teikyo University.

## Author Contributions

TF and SY designed the study and performed the research. TF, NA, CM, EF, KH, and SY analyzed the data. TF, NA, CM, TM, KH, and SY wrote the manuscript. All authors have reviewed the manuscript and contributed to the article and approved the submitted version.

## Conflict of Interest

The authors declare that the research was conducted in the absence of any commercial or financial relationships that could be construed as a potential conflict of interest.

## Publisher’s Note

All claims expressed in this article are solely those of the authors and do not necessarily represent those of their affiliated organizations, or those of the publisher, the editors and the reviewers. Any product that may be evaluated in this article, or claim that may be made by its manufacturer, is not guaranteed or endorsed by the publisher.

## Footnotes

1
https://www.hamamatsu.com/

2
https://imagej.nih.gov/ij/
