# Supplementary material for: Serotonergic Neurons in the Chick Brainstem Express Various Serotonin Receptor Subfamily Genes
Source: Front Physiol. 2022 Jan 17;12:815997. doi: 10.3389/fphys.2021.815997 (PMC8801614; doi:10.3389/fphys.2021.815997)
Supplement: Supplementary file 1 [file Data_Sheet_1.PDF]

Supplemental Table 1. Subject summary used in this study

| | chick #s | | | | | | |
|---|---|---|---|---|---|---|---|
| | #1 | #2 | #3 | #4 | #5 | #6 | #7 |
| probes | | | | | | | |
| ***TPH2*** | ○ | ○ | ○ | ○ | ○ | ○ | - |
| ***SERT*** | ○ | ○ | ○ | ○ | - | - | - |
| ***5-HTR1A*** | ○ | ○ | - | - | ○ | ○ | - |
| ***5-HTR1B*** | - | ○ | - | - | ○ | - | - |
| ***5-HTR1D*** | ○ | ○ | - | - | ○ | ○ | ○ |
| ***5-HTR1E*** | ○ | ○ | - | - | ○ | ○ | - |
| ***5-HTR1F*** | - | ○ | - | - | - | - | ○ |
| ***5-HTR2A*** | ○ | - | - | - | - | - | - |
| ***5-HTR2B*** | - | - | - | - | ○ | - | - |
| ***5-HTR2C*** | - | ○ | - | - | - | - | - |
| ***5-HTR3A*** | ○ | - | - | - | - | - | - |
| ***5-HTR4*** | - | - | - | - | ○ | - | - |
| ***5-HTR5A*** | ○ | ○ | - | - | - | ○ | ○ |
| ***5-HTR7*** | - | - | - | - | ○ | - | ○ |
